# X-Linked Osteogenesis Imperfecta Possibly Caused by a Novel Variant in *PLS3*

**DOI:** 10.3390/genes12121851

**Published:** 2021-11-23

**Authors:** Petar Brlek, Darko Antičević, Vilim Molnar, Vid Matišić, Kristina Robinson, Swaroop Aradhya, Dalibor Krpan, Dragan Primorac

**Affiliations:** 1 St. Catherine Specialty Hospital, 49210 Zabok/10000 Zagreb, Croatia; darko.anticevic@svkatarina.hr (D.A.); vilim.molnar@svkatarina.hr (V.M.); vid.matisic@svkatarina.hr (V.M.); 2 Laboratory of Neurooncology, Croatian Institute for Brain Research, School of Medicine, University of Zagreb, 10000 Zagreb, Croatia; 3 Faculty of Dental Medicine and Health, Josip Juraj Strossmayer University of Osijek, 31000 Osijek, Croatia; 4 Invitae, San Francisco, CA 94103, USA; kristina.robinson@invitae.com (K.R.); swaroop.aradhya@invitae.com (S.A.); 5 Polyclinic “K-Center”, for Internal Medicine, Gynecology, Radiology, Physical Medicine and Rehabilitation, 10 000 Zagreb, Croatia; dkrpan38@gmail.com; 6 Department of Biochemistry & Molecular Biology, The Pennsylvania State University, University Park, PA 16802, USA; 7 The Henry C. Lee College of Criminal Justice and Forensic Sciences, University of New Haven, West Haven, CT 06516, USA; 8 Medical School, University of Split, 21000 Split, Croatia; 9 School of Medicine, Josip Juraj Strossmayer University of Osijek, 31000 Osijek, Croatia; 10 Medical School, University of Rijeka, 51000 Rijeka, Croatia; 11 Medical School REGIOMED, 96 450 Coburg, Germany; 12 Medical School, University of Mostar, 88000 Mostar, Bosnia and Herzegovina

**Keywords:** osteogenesis imperfecta, X-linked osteoporosis, pathological fracture, PLS3, FN1, COL11A2

## Abstract

Osteogenesis imperfecta (OI) represents a complex spectrum of genetic bone diseases that occur primarily due to mutations and deletions of the *COL1A1* and *COL1A2* genes. Recent molecular studies of the network of signaling pathways have contributed to a better understanding of bone remodeling and the pathogenesis of OI caused by mutations in many other genes associated with normal bone mineralization. In this paper, a case of a rare X-linked variant of OI with a change in the gene encoding plastin 3—a protein important for the regulation of the actin cytoskeleton, is presented. A 16-year-old patient developed ten bone fractures caused by minor trauma or injury, including a compression fracture of the second lumbar vertebra during his lifetime. Next-generation sequencing analysis did not show pathologically relevant deviations in the *COL1A1* and *COL1A2* genes. Targeted gene analyses (Skeletal disorder panel) of the patient, his father, mother and sister were then performed, detecting variants of uncertain significance (VUS) for genes *PLS3*, *FN1* and *COL11A2*. A variant in the *PLS3* gene were identified in the patient, his mother and sister. Since the *PLS3* gene is located on the X chromosome, the mother and sister showed no signs of the disease. Although the variant in the *PLS3* gene (c.685G>A (p.Gly229Arg)) has not yet been described in the literature, nor is its pathogenicity known, clinical findings combined with genetic testing showed that this variant may explain the cause of X-linked OI in our patient. This rare case of the *PLS3* variant of X-linked OI might point to a novel target for personalized therapy in patients with this severe disease.

## 1. Introduction

Osteogenesis imperfecta (OI) is a clinically and genetically heterogeneous group of diseases that is inherited in an autosomal dominant, autosomal recessive, and X-linked manner [1,2]. This hereditary skeletal dysplasia manifests with three main clinical hallmarks: bone fragility ("brittle bone disease"), skeletal deformities and growth deficiency [2,3]. The clinical presentation of OI is variable in its severity. In addition to the skeletal findings, it can affect multiple organ systems and cause secondary complications. The most common secondary features include macrocephaly and dental abnormalities, blue sclerae, hearing loss, respiratory and cardiopulmonary complications [4]. The traditional types of OI (types I-IV) are inherited in an autosomal dominant manner and encompass about 80–85% of OI cases [2]. These cases are caused by pathogenic variants in exons of the genes that encode type I collagen, which is essential for normal extracellular matrix (ECM) function [5]. Previous studies have shown that in addition to pathogenic variants within exons, intronic region variants can cause splice-defective COL1A1 transcripts that also manifest with symptoms of OI [6,7]. Dysfunction of the molecular mechanisms that regulate bone mineralization, formation of ECM and normal osteoblast differentiation plays a key role in the pathogenesis of OI [2,8]. Osteocytes control bone turnover by regulating the activity of both osteoclasts and osteoblasts, and they enable constant remodeling of the extracellular matrix. The imbalance of osteoclast-mediated bone resorption and osteoblast-mediated bone formation can result in either loss or gain of bone mass [9]. In recent years, it has become clear that bone remodeling is regulated by several signaling pathways. The main pathways included in the regulation of bone formation are the Hedgehog (HH), parathyroid hormone-related peptide (PTHrP), fibroblast growth factor (FGF), C-type natriuretic peptide (CNP), transforming growth factor-beta (TGFβ), bone morphogenetic protein (BMP), Notch, WNT and osteocyte mechanosensing pathway [10,11,12]. Any disruption of genes whose protein products are involved in the molecular network of these signaling pathways can cause changes in bone remodeling and poor mineralization. Dysregulation of these pathways disrupts the mechanisms that control skeletal strength and integrity, leading to bone fragility associated with reduced bone mass [11].

Due to the discovery of a large number of new genes involved in the pathogenesis of OI, new classifications of this disease have been made based not only on the clinical but also on the molecular characteristics of OI. A classification by Marini et al. based on the molecular etiopathogenesis of the disease classifies OI by defects in collagen synthesis, bone mineralization, collagen modification and processing, and defects in osteoblast differentiation [2]. The majority of OI cases (type I-IV) are associated with reduced production of normal type I collagen or the synthesis of abnormal collagen as a result of pathogenic variants in *COL1A1* and *COL1A2* genes [3]. Pathogenic variants in the *IFITM5* (OI type V) and *SERPINF1* (OI type VI) genes are responsible for deficiencies in bone mineralization [13]. Among the genes responsible for OI with deficits in collagen modification and processing are *CRTAP* (type VII), *LEPREI* (type VIII), *PPIB* (type IX), *SERPINH1* (type X), *FKBP10* (type XI) and *BMP1* (type XII). Pathogenic variants in genes *SP7* (type XIII), *TMEM38B* (type XIV), *WNT1* (type XV), *CREB3L1* (type XVI), *SPARC* (type XVII) and *MBTPS2* (type XVIII) cause defects in osteoblast differentiation [2].

Recently discovered cases of OI have been associated with pathogenic variants in an X-chromosome gene coding for plastin 3 (PLS3). The clinical presentation in hemizygous men matched the presentation of classical OI and was variable in heterozygous women [14]. The main role of PLS3 is in F-actin-binding, which consequently suggests that PLS3 participates in all processes dependent on F-actin dynamics, such as cell motility, cell division, focal adhesion, endocytosis, neurotransmission, vesicle trafficking, axonal local translation, and intracellular calcium PLS3-dependent processes [15,16]. Plastins are proteins with a single polypeptide chain composed of two tandem repeats of actin-binding domains (ABD1 and ABD2). Each ABD is assembled from two tandem calponin-homology (CH) domains (CH1 and CH2 in ABD1, and CH3 and CH4 in ABD2) [17]. The binding of each ABD to two separate actin filaments promotes the formation of a bundle resulting in distinct F-actin organization [16].

In the present study, a novel *PLS3* variant in a nonconsanguineous family of a proband with X-linked OI was detected and potential link between this variant of plastin 3 and osteogenesis imperfecta was reviewed.

## 2. Materials and Methods

### 2.1. Subjects

A 16-year-old patient (proband), who complained of pain, pathological fractures and patellar subluxations that arose due to a moderate valgus of the distal femur, was admitted to St. Catherine’s Special Hospital. The patient was accompanied by his father, mother and sister, who had no symptoms related to diseases of the locomotor system and who were all included in the study. All participants involved in this study signed an informed consent form.

### 2.2. Clinical Examination

Clinical data were collected, including fracture history, height, weight, growth speed and family history. Additionally, the sclera, teeth, hearing and musculoskeletal system were checked upon clinical examination. Blood analysis was performed to determine serum calcium, inorganic phosphates, osteocalcin and vitamin D (25-OH) in all patients. Moreover, biochemical findings of creatinine and deoxypyridinoline were obtained from urine. The results of the analysis were interpreted according to the reference intervals of the Laboratory of the Special Hospital St. Catherine. Bone densitometry was performed in the whole family, which included the measurement of bone density of the proximal femur and spine, whose reference values were validated according to the Croatian population. The proband received high-dose vitamin D therapy (25,000 IU/day) for three months. To evaluate the effect of the therapy, we measured markers of bone remodeling and bone mineral density (BMD) measured by densitometry.

### 2.3. Genetic Testing

Genomic DNA was isolated from the patient’s blood sample and subjected to clinical next-generation sequencing using a multi-gene panel. Invitae Skeletal Disorders Panel includes sequence analysis and deletion/duplication testing of 320 genes was conducted (Table 1). All target genes were sequenced to a minimum depth of ≥50× and an average of 350×. Sequence reads were aligned with the reference genome (GRCh37) and single nucleotide variants (SNVs) were called from coding sequences and 20 bp of flanking intronic sequences. Promoters and other non-coding regions were not included. Exon-level copy number (deletions and duplications) and other types of non-SNV variants were identified using validated algorithms [18,19].

### 2.4. Bioinformatics Analysis of Gene Variants of Unknown Significance

Bioinformatics software Sorting Intolerant to Tolerant (SIFT), Polymorphism Phenotyping v2 (PolyPhen-2) and Align-GVGD were used to predict the possible pathogenicity of the gene variants found in proband’s sample. SIFT (https://sift.bii.a-star.edu.sg/) is a tool that predicts the possible impact of an amino acid substitution based on sequence homology and the physical properties of amino acids. PolyPhen-2 (http://genetics.bwh.harvard.edu/pph2/) predicts whether an amino acid substitution affects protein function by comparing the physical and chemical properties of amino acids. Align-GVGD (http://agvgd.hci.utah.edu/) is a freely available program that combines the biophysical characteristics of amino acids and protein multiple sequence alignment to predict possible pathogenicity of gene variants.

## 3. Results

### 3.1. Phenotypes of the Patients

**Figure 1:**
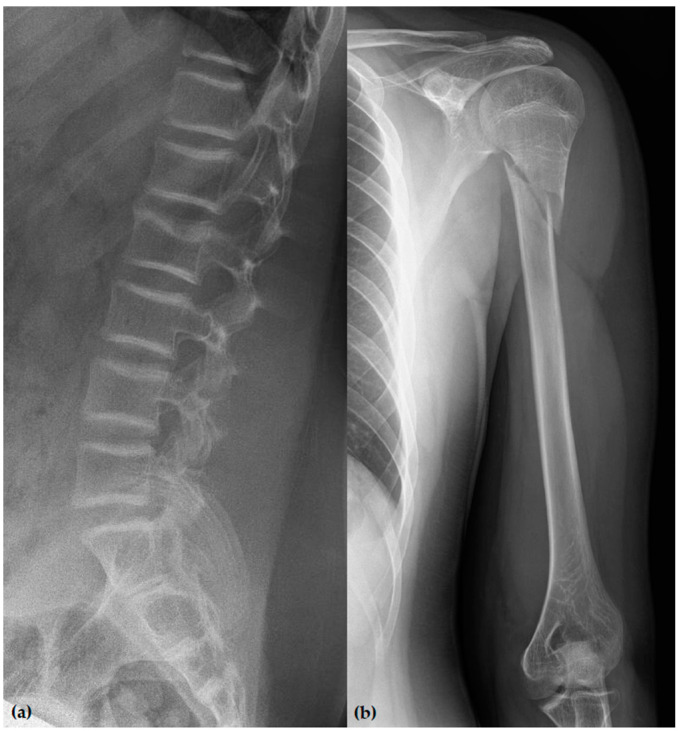
(**a**) Profile lumbar spine radiograph showing a compression fracture of the L2 vertebral body. The superior endplate is compressed posteriorly, with minor loss of vertebral body height corresponding to grade 1 by Genant classification. (**b**) Anteroposterior radiograph of the left upper arm showing a spiral fracture of the proximal third of the humerus.

The proband, a 15-year-old boy (164 cm, 65 kg, BMI = 24.2 kg/m^2^), was the first child of a nonconsanguineous family. He was delivered vaginally at term with a birth weight of 3500 g. In the maternity ward, he had neonatal jaundice and phototherapy was performed. His psychomotor development was normal. He suffered his first low-trauma fracture (fifth metatarsal bone of his right foot) at the age of 2 years and ten months. Since then, he had experienced a total of 10 fractures, including a fracture of the neck of the left humerus, the radius metaphysis and a compression fracture of the L2 vertebra (Figure 1).

During the physical examination, impaired growth was determined (3rd–15th percentile), while sclerae, dentition and joint laxity were normal. Although the hearing loss was initially observed at age 3, the most recent hearing test showed a normal tympanogram and tonal audiogram. Radiographic images showed inadequate bone mineralization, while densitometry revealed reduced bone density in favor of osteoporosis. Laboratory findings showed decreased levels of vitamin D (25-OH). The serum concentration was normalized after administering an increased dose of vitamin D (25,000 IU). The proband had normal inorganic phosphates, creatinine, ALP, ALT and deoxypyridinoline concentrations with an increased serum concentration of osteocalcin and calcium. He had low BDMs at the lumbar spine of 0.587 g/cm^2^ (L1-L4 T score −4.3) and left hip of 0.604 g/cm^2^ (T score −2.8). The proband’s 14-year-old sister from the same parents was healthy (163 cm, 89 kg, BMI = 33.5 kg/m^2^) with normal hearing, sclerae, dentition and joint laxity. Laboratory findings showed normal serum calcium and osteocalcin levels with significantly reduced inorganic phosphates and vitamin D (25-OH). She had BDMs at the lumbar spine of 0.990 g/cm^2^ (L1-L4 T score −0.5) and left hip of 0.971 g/cm^2^ (T score 0.2), which indicate initial osteoporotic changes. The mother of the proband was healthy (155 cm, 70 kg, BMI = 29.1kg/m^2^). She had normal hearing, sclerae, dentition and joint laxity. Laboratory findings showed normal serum calcium levels, osteocalcin and inorganic phosphates with significantly reduced vitamin D (25-OH). She had BDMs at the lumbar spine of 0.897 g/cm^2^ (L1-L4 T score −1.4) and left hip of 0.903 g/cm^2^ (T score −0.3), which indicate initial osteoporotic changes. The mother and her siblings had not yet experienced bone fractures. The proband’s father and his parents were healthy with no history of fractures. More clinical features of the whole family with results of bone remodeling markers measured on 1 September 2021, are shown in Table 2.

### 3.2. Genetic Findings

**Figure 2:**
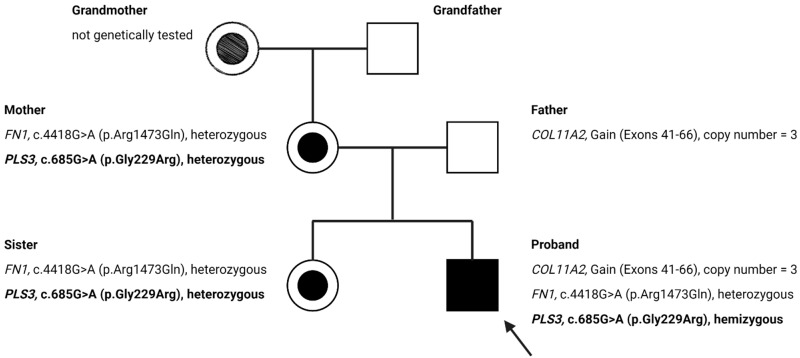
The pedigree of the family with X-linked osteogenesis imperfecta (OI) and variants of unknown significance (VUS) for the *PLS3*, *NF1*, and *COL11A2* genes. The proband is marked with an arrow. The proband’s sister, mother and her siblings had no history of fractures. Created with BioRender.com (accessed on 10 November 2021).

Multiplex ligation-dependent probe amplification (MLPA) analysis and next-generation sequencing analysis performed on the *COL1A1* and *COL1A2* genes excluded pathogenic variants. Subsequent analysis performed using a multi-gene skeletal disorder panel on the proband’s blood sample identified variants of uncertain significance (VUS) in *PLS3*, *FN1* and *COL11A2* (Figure 2). These variants were not present in the Genome Aggregation Database (gnomAD), the Exome Aggregation Consortium (ExAC), or Invitae’s in-house variant database. 

The c.4418G>A (p.Arg1473Gln) variant in exon 28 of the *FN1* gene was heterozygous in both the mother and the patient, thus reducing the likelihood that it is the cause of the disease. Additionally, we found an amplification of exons 41–66 of the *COL11A2* gene in the proband and his healthy father. The exact location of this copy-number change is unknown. The analysis from Invitae suggests that the 5’ breakpoint is likely in intron 40, but the 3’ boundary is difficult to determine as it likely exists beyond the end of the gene.

A c.685G>A (p.Gly229Arg) variant in the X-linked *PLS3* gene was identified in the patient, his mother and his sister. This variant has not yet been described in the literature, nor is its pathogenicity known. Furthermore, we confirmed the absence of this variant in the proband’s maternal grandfather, suggesting that the variant either occurred de novo in the proband’s mother or she may have inherited it from the proband’s grandmother.

Along with these observations, our clinical findings indicate that this variant may explain the X-linked OI in the proband. Since the *PLS3* gene is located on the X chromosome, the mother and sister were not expected to show pathological fractures if this variant were definitively pathogenic. The results of the genetic analysis performed in this family has led to the discovery of an apparently novel variant in the *PLS3* gene, for which the available evidence indicates a favorable likelihood of pathogenicity (Figure 2).

### 3.3. Bioinformatics Analysis

**Figure 3:**
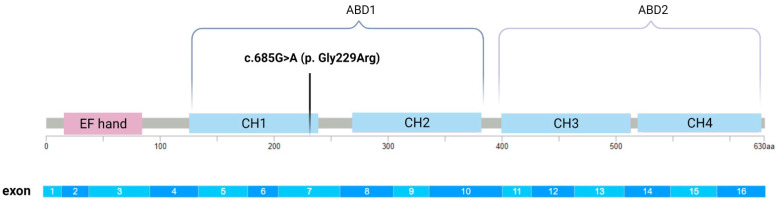
Molecular structure of the PLS3 protein with an indicated mutation in exon 7, which is a part of the CH1 (calponin-homology 1) domain. Created with BioRender.com (accessed on 10 November 2021).

Algorithms developed to predict the effect of missense changes on protein structure and function showed results in favor of the pathogenicity of the newly discovered variant of the *PLS3* gene (Figure 3).

The new variant in the *PLS3* gene was characterized as “deleterious” by SIFT software, which predicts whether amino acid substitution affects protein function. A similar result was shown by the bioinformatics tool PolyPhen-2, which classified the change of *PLS3* gene (c.685G>A (p.Gly229Arg)) as “probably harmful”.

Align-GVGD, a web-based program that combines the biophysical characteristics of amino acids and protein multiple sequence alignments, classified the new variant of the *PLS3* gene as “Class C15”. Prediction groups form a spectrum from C0 to C65, with C0 least likely to interfere with protein function and C65 most likely.

### 3.4. The Effect of Vitamin D Treatment

Hypovitaminosis was successfully corrected, and there was an improvement in bone density after three months of therapy with increased doses of vitamin D (25,000 IU/day). Bone densitometry confirmed the increase in BMD measured on 1 September 2021, compared to BMD on 20 May 2021 on both the femur (F1 BDM = 0.604; F2 BDM = 0.689) and the spine (S1 BDM = 0.587; S2 BDM = 0.622) (Table 2).

## 4. Discussion

We identified a Croatian family with X-linked OI caused by a novel missense variant in the *PLS3* gene (c.685G>A (p.Gly229Arg)). The proband presented with low bone mass, frequent pathological fractures and occasional subluxations of the patella. His mother and sister were healthy without previous fractures; however, the densitometry findings revealed initial osteoporotic changes. All of the family members had normal hearing, sclerae, dentition and joint laxity. These findings suggest that the variant in *PLS3*, described now for the first time, may have an impact on the process of bone formation or mineralization, while its role in odontogenesis and the processes associated with the formation of other connective tissue is not clinically noticeable. Interestingly, Hu et al. in their case of X-linked OI showed an entire family that had characteristic blue sclera, while the proband and his family did not have the stated characteristic. Additionally, in their study, it was stated that the mother, as the carrier of the mutation, had normal BMD, while in this case the proband’s sister and mother had reduced BMD [14]. Densitometric findings indicated initial osteoporotic changes visible on the bones of the spine and proximal femur. Such findings suggest a diverse range of clinical phenotypes in women as the process of X chromosome inactivation is random and leads to mosaicism [20].

Studies indicate that pathogenic variants in the *PLS3* gene, which encodes plastin 3, play a major role in bone metabolism and lead to severe early osteoporosis [21]. Different variants in *PLS3*, which is ubiquitously expressed in solid tissues, lead to decreased bone mineral density [22]. Previous findings suggest that the majority of the OI-linked *PLS3* pathogenic variants are either loss-of-function changes (nonsense or frameshift varaints) which rarely result in translated protein products due to nonsense-mediated mRNA decay [16,23]. Separately, a rare single nucleotide polymorphism of the *PLS3* gene was reported in association with osteoporosis in postmenopausal women [24]. The current identified X-linked *PLS3* actin bundling-deficient mutation (L478P) that produces a full-length protein disables actin-binding in the ABD2 and thus prevents F-actin bundling. The bundling-deficient PLS3 fails to co-localize with any F-actin structures in cells despite preserved F-actin binding through a non-mutation-bearing ABD [25]. Our results indicate that the Gly229Arg missense change in exon 7, which encodes a CH1 domain that is a key part of ABD1, may thus cause actin-binding disorders. Based on literature reports, we believe that disease-causing variants in the *PLS3* gene are generally loss-of-function. However, there is currently not enough evidence available to determine whether p.Gly229Arg is a loss-of-function variant.

Different variants in *PLS3* have shown differences in distribution between lamellipodia and focal adhesions [16]. Studies on the chicken homolog of the *PLS3* gene have shown that the function of its protein product can be linked to mechanosensitivity of osteocytes [26]. Dendrites are the most mechanosensitive part of the osteocyte and they are indicators of overall osteocyte mechanosensitivity [27]. Increased *PLS3* expression was observed during osteoblast maturation and within osteocyte dendritic processes indicating its role in bone morphogenesis and remodeling [25]. Although other examined mutations that produce a full-length protein have fully retained F-actin bundling ability, it is shown that they have defects in Ca^2+^ sensitivity. While wild-type PLS3 was distributed equally in lamellipodia and focal adhesions, the Ca^2+^-hyposensitive PLS3 was localized exclusively at focal adhesions. On the other hand, the Ca^2+^-hypersensitive PLS3 mutants were bound to lamellipodia. These findings unveiled that severe osteoporosis can be caused by a mutational disruption of the Ca^2+^-controlled PLS3’s cycling between lamellipodia and focal adhesions [16]. Additionally, it is possible that the *PLS3* mutation we found in exon 7 (c.685G>A), which replaces the amino acid glycine with arginine, changes the conformation of PLS3 itself and consequently leads to hypersensitivity or hyposensitivity of the PLS3 protein to calcium. Such a change in calcium sensitivity would lead to misregulation of actin cytoskeleton remodeling and consequently to OI symptoms as found in our patient. Although the exact mechanism of pathogenesis of the novel *PLS3* variant we have described here remains unknown, the genetic analysis in the family, absence in population genomic databases, and the in silico predictions suggest that it is very likely a pathogenic variant that causes X-linked OI unrelated to collagenopathies.

Discoveries in the field of bone development and actin cytoskeleton reorganization provide a better understanding of mechanisms by which plastin 3 causes OI [28]. The leading hypotheses include insufficient mineralization by osteoblasts, dysregulation of osteocyte mechanosensing, and increased bone resorption by osteoclasts [16,23,27,29,30]. Previous findings showed that PLS3 actin-bundling activity, as well as finely tuned Ca^2+^ regulation, are essential for proper bone formation [16]. Different localization of PLS3 within cells is altered by sensitivity to Ca^2+^, which suggests that fine regulation of PLS3 by Ca^2+^ is critical for bone formation, as its imbalance in either direction results in OI [25]. Studies on animal models have indicated the importance of PLS3 in bone tissue development and preservation of bone architecture [26]. The mouse knockout model for *PLS3* showed decreased bone strength and osteoporosis, while *PLS3* knockdown in zebrafish manifests in muscular and skeletal abnormalities [28,30]. In contrast to classical OI caused by *COL1A1* and *COL1A2* variants or a mutation in the *IFITM5* gene (OI type V), which result in hypermineralized bone matrix, defects in PLS3 cause significant hypomineralization of the bone matrix [31,32,33]. Such findings were confirmed by densitometry in our patients (mother, sister and proband), whose bones showed a loss of bone density in support of the diagnosis of osteoporosis. A recent study has proposed a role for PLS3 in osteoclast activity through the regulation of podosomes by nuclear factor κB (NFκB) signaling [30]. Receptor activator of nuclear factor κB ligand (RANKL) signaling inhibits osteoblastic differentiation mainly through activating NFκB as well as inhibiting the β-catenin synthesis and promoting osteoclastogenesis [12]. Since PLS3 represents the major plastin isoform in osteocytes, it could contribute to both osteogenesis and osteolysis [34]. The findings that Ca^2+^ is involved in the redistribution of PLS3 from focal adhesions to the leading edge represent a strong link between the activities of PLS3 and the machinery thought to drive bone mechanosensing and reorganization [25].

In recent years, the genetic range of diseases associated with osteoporosis has expanded widely and, so far, at least 24 genes have been identified to cause OI [11]. Mechanistic studies in vitro and preclinical mouse models have demonstrated defects in type I collagen processing and crosslinking, post-translational modifications, folding, procollagen transport from rough ER to the Golgi or collagen secretion and structure [11,35]. Moreover, some forms of OI associated with collagen type I deposition and mineralization are caused by mutations in *SERPINF1* or *IFITM5*, while mutations in *WNT1* or *SP7* are linked to inhibition of chondrocyte differentiation and stimulation of osteoblast differentiation [36,37,38,39]. Other diseases associated with disordered bone formation, in which collagen processing is not affected, have disrupted cellular signaling via the WNT, the RANKL-RANK and the NOTCH2 signaling pathways, which are important in the regulation of bone resorption and formation [11,40,41,42]. The understanding of these pathways through the study of rare bone diseases has opened up new possibilities of specific therapeutic agents for the treatment of common osteoporosis. Many rare fragility disorders remain insufficiently understood and hence drug targets remain undiscovered for future drug development. Acquired bone fragility conditions such as cytokine and glucocorticoid-induced as well as postmenopausal osteoporosis are far more common and new, pathway-specific treatments are still needed [11].

Today, there are numerous therapeutic options for the treatment of OI, including bisphosphonates, denosumab, teriparatide, sclerostin inhibitory antibody, transforming growth factor-beta inhibition, orthopedic management of OI and, among the latest therapies, the use of stem cells [4,43]. Bisphosphonates are the basis of pharmacological treatment and act by inhibiting osteoclast activity and enhancing bone resorption. Current evidence demonstrates that bisphosphonates increase bone mineral density in children and adults with OI and also reduce the risk of fractures [44]. They are most commonly used in pediatric patients because, during growth, they favorably affect the fusion of the vertebrae after compression fractures. However, when using them, care should be taken to avoid side effects that include the acute phase of the infusion reaction and transient hypocalcemia [4,45]. In our case, we showed that high doses of vitamin D improved bone density after three months as evidenced by densitometry findings performed on the proximal femur and lumbar spine. Among the drugs for OI, biological drugs like denosumab are being investigated today. This drug is an antibody for RANKL and inhibits osteoclast differentiation and function [46]. Additionally, like bisphosphonates, it acts on osteoclasts in order to inhibit bone resorption. Several studies have shown that denosumab treatment improves bone mineral density in patients with OI [4]. Another inhibitory antibody (sclerostin) has an inhibitory effect on bone formation via the canonical WNT signaling pathway. Dysregulation of sclerostin expression causes skeletal disorders characterized by loss of bone mass [47]. Romosozumab is a humanized monoclonal antibody that inhibits bone resorption by inhibiting sclerostin and promoting bone formation [48]. The parathyroid hormone analog (teriparatide) induces bone anabolism and stimulates bone formation before it enhances bone resorption in adults with type I OI [49]. Drugs that inhibit transforming growth factor-beta (TGFβ) act on TGFβ signaling, which is extremely important for the formation of the skeleton [50]. Fresolimumab is one of the TGFβ inhibitors and is currently being studied in adult patients with OI [4]. By performing a complex two-part operation in the severe form of OI type III, Jeleč et al. demonstrated that, aside from drug therapy, personalized surgical treatment has an important role in treating OI patients [51]. Recently, mesenchymal stem cells (MSCs) have been suggested as an ideal tool for bone and cartilage regeneration [52]. Research on the treatment of OI is also developing in the direction of MSC transplants. This form of therapy is a personalized treatment that starts before birth or as soon as possible after birth. In this way, it is possible to prevent fractures, which is not possible with any other therapy available today [29].

The limitation of the study was the small number of people we could test for this variant of the PLS3 gene and relate to the clinical phenotype since we found a novel variant never before described in the literature.

## 5. Conclusions

The normal process of bone remodeling and mineralization is extremely important for the formation of sufficiently strong bones. To improve the treatment of patients with bone dysplasia, further studies of the molecular substrate involved in the regulation of signaling pathways that control bone remodeling are needed. Our discovery of a novel missense variant in the *PLS3* gene that is segregating with disease in a Croatian family and present in a hemizygous state in an affected male proband suggests a link to X-linked OI. The protein product of *PLS3* participates in the reorganization of the actin cytoskeleton contributes to a better understanding of the involvement of this gene in the pathogenesis of X-linked OI. Future research of the role of plastin 3 in the function of bone remodeling regulation by osteoblasts and osteoclasts will shed light on potential molecular targets in personalized therapy.

## Figures and Tables

**Table 1:** A complete list of genes analyzed, including the relevant gene transcripts.

GENE	TRANSCRIPT	GENE	TRANSCRIPT	GENE	TRANSCRIPT	GENE	TRANSCRIPT
*ACAN*	NM_013227.3	*CDKN1C*	NM_000076.2	*DHCR24*	NM_014762.3	*C2CD3*	NM_015531.5
*ACP5*	NM_001111035.2	*CDT1*	NM_030928.3	*DIP2C*	NM_014974.2	*CA2*	NM_000067.2
*ACVR1*	NM_001105.4	*CENPJ*	NM_018451.4	*DLL3*	NM_016941.3	*CANT1*	NM_138793.3
*ADAMTS10*	NM_030957.3	*CEP120*	NM_153223.3	*DLX3*	NM_005220.2	*CASR*	NM_000388.3
*ADAMTS17*	NM_139057.3	*CEP135*	NM_025009.4	*DMRT2*	NM_006557.6	*CCDC8*	NM_032040.4
*AFF4*	NM_014423.3	*CEP152*	NM_014985.3	*DNA2*	NM_001080449.2	*CDC45*	NM_001178010.2
*AGA*	NM_000027.3	*CEP63*	NM_025180.3	*DONSON*	NM_017613.3	*CDC6*	NM_001254.3
*AGPS*	NM_003659.3	*CFAP410*	NM_004928.2	*DVL1*	NM_004421.2	*FN1*	NM_212482.2
*AIFM1*	NM_004208.3	*CHST14*	NM_130468.3	*DVL3*	NM_004423.3	*FTO*	NM_001080432.2
*ALPL*	NM_000478.5	*CHST3*	NM_004273.4	*DYM*	NM_017653.3	*FUCA1*	NM_000147.4
*AMER1*	NM_152424.3	*CHUK*	NM_001278.4	*DYNC2H1*	NM_001080463.1	*FZD2*	NM_001466.3
*ANKH*	NM_054027.4	*CLCN7*	NM_001287.5	*DYNC2LI1*	NM_016008.3	*GALNS*	NM_000512.4
*ANO5*	NM_213599.2	*COG1*	NM_018714.2	*EBP*	NM_006579.2	*GALNT3*	NM_004482.3
*ARCN1*	NM_001655.4	*COL10A1*	NM_000493.3	*EIF2AK3*	NM_004836.6	*GDF5*	NM_000557.4
*ARSB*	NM_000046.3	*COL11A1*	NM_001854.3	*ESCO2*	NM_001017420.2	*GDF6*	NM_001001557.2
*ARSE*	NM_000047.2	*COL11A2*	NM_080680.2	*EVC*	NM_153717.2	*GHR*	NM_000163.4
*ASCC1*	NM_001198800.2	*COL1A1*	NM_000088.3	*EVC2*	NM_147127.4	*GHRHR*	NM_000823.3
*ASPM*	NM_018136.4	*COL1A2*	NM_000089.3	*EXOC6B*	NM_001321729.1	*GHSR*	NM_198407.2
*ATR*	NM_001184.3	*COL27A1*	NM_032888.3	*EXOSC2*	NM_014285.6	*CSGALNACT1*	NM_001130518.1
*B3GALT6*	NM_080605.3	*COL2A1*	NM_001844.4	*EXT1*	NM_000127.2	*CSPP1*	NM_024790.6
*B3GAT3*	NM_012200.3	*COL9A1*	NM_001851.4	*EXT2*	NM_207122.1	*CTSA*	NM_000308.3
*B4GALT7*	NM_007255.2	*COL9A2*	NM_001852.3	*EXTL3*	NM_001440.3	*CTSK*	NM_000396.3
*BGN*	NM_001711.5	*COL9A3*	NM_001853.3	*FAM20C*	NM_020223.3	*CUL7*	NM_014780.4
*BMP1*	NM_006129.4	*COMP*	NM_000095.2	*FAM46A*	NM_017633.2	*CWC27*	NM_005869.3
*BMP2*	NM_001200.3	*CREB3L1*	NM_052854.3	*FAR1*	NM_032228.5	*DDR2*	NM_006182.2
*BMPER*	NM_133468.4	*CRTAP*	NM_006371.4	*FBN1*	NM_000138.4	*DDRGK1*	NM_023935.2
*BMPR1B*	NM_001203.2	*CSF1R*	NM_005211.3	*FGF23*	NM_020638.2	*IFT43*	NM_052873.2
*IFT52*	NM_001303458.2	*IFT122*	NM_052985.3	*LIG4*	NM_002312.3	*MYH3*	NM_002470.3
*IFT57*	NM_018010.3	*IFT140*	NM_014714.3	*LMNA*	NM_170707.3	*MYO18B*	NM_032608.6
*IFT74*	NM_001099222.1	*IFT172*	NM_015662.2	*LMX1B*	NM_002316.3	*NAGLU*	NM_000263.3
*IFT80*	NM_020800.2	*IDUA*	NM_000203.4	*LONP1*	NM_004793.3	*NANS*	NM_018946.3
*IFT81*	NM_014055.3	*IFITM5*	NM_001025295.2	*LOXL3*	NM_032603.3	*NBAS*	NM_015909.3
*IGF1*	NM_000618.4	*IFT122*	NM_052985.3	*LRP4*	NM_002334.3	*NEK1*	NM_012224.2
*IGF2*	NM_000612.5	*IFT140*	NM_014714.3	*LRP5*	NM_002335.3	*NEU1*	NM_000434.3
*IHH*	NM_002181.3	*IFT172*	NM_015662.2	*LRRK1*	NM_024652.4	*NKX3-2*	NM_001189.3
*IMPAD1*	NM_017813.4	*IDUA*	NM_000203.4	*LTBP2*	NM_000428.2	*NOG*	NM_005450.4
*FGF9*	NM_002010.2	*IFITM5*	NM_001025295.2	*LTBP3*	NM_001130144.2	*NOTCH2*	NM_024408.3
*FGFR1*	NM_023110.2	*PCYT1A*	NM_005017.3	*MAFB*	NM_005461.4	*NPPC*	NM_024409.3
*FGFR2*	NM_000141.4	*PDE4D*	NM_001104631.1	*MAN2B1*	NM_000528.3	*NPR2*	NM_003995.3
*FGFR3*	NM_000142.4	*PEX5*	NM_001131025.1	*MANBA*	NM_005908.3	*NPR3*	NM_000908.3
*FIG4*	NM_014845.5	*PEX7*	NM_000288.3	*MAP3K7*	NM_145331.2	*NSDHL*	NM_015922.2
*FKBP10*	NM_021939.3	*PGM3*	NM_001199917.1	*MATN3*	NM_002381.4	*NSMCE2*	NM_173685.2
*FLNA*	NM_001456.3	*PISD*	NM_001326411.1	*MBTPS2*	NM_015884.3	*NXN*	NM_022463.4
*FLNB*	NM_001457.3	*PKDCC*	NM_138370.2	*SH3PXD2B*	NM_001017995.2	*OBSL1*	NM_015311.2
*MCM5*	NM_006739.3	*PLK4*	NM_014264.4	*SLC17A5*	NM_012434.4	*OCRL*	NM_000276.3
*MCPH1*	NM_024596.4	*PLOD2*	NM_182943.2	*SLC26A2*	NM_000112.3	*ORC1*	NM_004153.3
*MEOX1*	NM_004527.3	*PLS3*	NM_005032.6	*SLC35D1*	NM_015139.2	*ORC4*	NM_002552.4
*MESP2*	NM_001039958.1	*POC1A*	NM_015426.4	*SLC39A13*	NM_152264.4	*ORC6*	NM_014321.3
*MGP*	NM_000900.3	*POLR1A*	NM_015425.4	*SLCO2A1*	NM_005630.2	*OSTM1*	NM_014028.3
*MMP13*	NM_002427.3	*POP1*	NM_015029.2	*SLCO5A1*	NM_030958.2	*P3H1*	NM_022356.3
*MMP14*	NM_004995.3	*POR*	NM_000941.2	*SMAD4*	NM_005359.5	*P4HB*	NM_000918.3
*MMP2*	NM_004530.5	*PPIB*	NM_000942.4	*SMARCAL1*	NM_014140.3	*PAM16*	NM_016069.9
*MMP9*	NM_004994.2	*PPP3CA*	NM_000944.4	*SNRPB*	NM_198216.1	*PAPSS2*	NM_001015880.1
*MNX1*	NM_005515.3	*PRKAR1A*	NM_002734.4	*SNX10*	NM_001199835.1	*PCGF2*	NM_007144.2
*GJA1*	NM_000165.4	*PTDSS1*	NM_014754.2	*SOX9*	NM_000346.3	*PCNT*	NM_006031.5
*GLB1*	NM_000404.2	*PTH1R*	NM_000316.2	*SP7*	NM_001173467.2	*TRMT10A*	NM_152292.4
*GMNN*	NM_015895.4	*PTHLH*	NM_198965.1	*SPARC*	NM_003118.3	*TRPS1*	NM_014112.4
*GNAS*	NM_000516.5	*PTPN11*	NM_002834.3	*SQSTM1*	NM_003900.4	*TRPV4*	NM_021625.4
*GNE*	NM_001128227.2	*PYCR1*	NM_006907.3	*SRCAP*	NM_006662.2	*TTC21B*	NM_024753.4
*GNPAT*	NM_014236.3	*RAB33B*	NM_031296.2	*SUCO*	NM_014283.4	*TUBGCP6*	NM_020461.3
*GNPTAB*	NM_024312.4	*RBBP8*	NM_002894.2	*SULF1*	NM_001128205.1	*TYROBP*	NM_003332.3
*GNPTG*	NM_032520.4	*RECQL4*	NM_004260.3	*TAB2*	NM_015093.5	*VAC14*	NM_018052.3
*GNS*	NM_002076.3	*RIPPLY2*	NM_001009994.2	*TAPT1*	NM_153365.2	*VPS33A*	NM_022916.4
*GORAB*	NM_152281.2	*RMRP*	NR_003051.3	*TBCE*	NM_003193.4	*WDR19*	NM_025132.3
*GPC6*	NM_005708.3	*RNU4ATAC*	NR_023343.1	*TBX15*	NM_152380.2	*WDR34*	NM_052844.3
*GPX4*	NM_001039848.2	*SFRP4*	NM_003014.3	*TBX3*	NM_005996.3	*WDR35*	NM_001006657.1
*GSC*	NM_173849.2	*INPPL1*	NM_001567.3	*TBX5*	NM_000192.3	*WDR60*	NM_018051.4
*GUSB*	NM_000181.3	*JAG1*	NM_000214.2	*TBX6*	NM_004608.3	*WISP3*	NM_003880.3
*GZF1*	NM_022482.4	*KAT6B*	NM_012330.3	*TBXAS1*	NM_001061.4	*WNT1*	NM_005430.3
*HES7*	NM_032580.3	*KIAA0586*	NM_001244189.1	*TCIRG1*	NM_006019.3	*WNT3*	NM_030753.4
*HGSNAT*	NM_152419.2	*KIAA0753*	NM_014804.2	*TCTEX1D2*	NM_152773.4	*WNT3A*	NM_033131.3
*HPGD*	NM_000860.5	*KIF22*	NM_007317.2	*TCTN3*	NM_015631.5	*WNT5A*	NM_003392.4
*HSPG2*	NM_005529.6	*KL*	NM_004795.3	*TGFB1*	NM_000660.5	*XRCC4*	NM_022406.3
*HYAL1*	NM_153281.1	*KMT2A*	NM_001197104.1	*TMEM165*	NM_018475.4	*XYLT1*	NM_022166.3
*IARS2*	NM_018060.3	*LARP7*	NM_016648.3	*TMEM38B*	NM_018112.2	*XYLT2*	NM_022167.3
*ICK*	NM_016513.4	*LBR*	NM_002296.3	*TNFRSF11A*	NM_003839.3	*SGSH*	NM_000199.3
*IDS*	NM_000202.6	*LEMD3*	NM_014319.4	*TNFRSF11B*	NM_002546.3	*ROR2*	NM_004560.3
*IDUA*	NM_000203.4	*LFNG*	NM_001040167.1	*TNFSF11*	NM_003701.3	*RSPRY1*	NM_133368.2
*IFITM5*	NM_001025295.2	*LIFR*	NM_002310.5	*MSX2*	NM_002449.4	*RTTN*	NM_173630.3
*SEC24D*	NM_014822.3	*TRAPPC2*	NM_001011658.3	*SERPINH1*	NM_001235.3	*RUNX2*	NM_001024630.3
*TRIP11*	NM_004239.4	*TREM2*	NM_018965.3	*SERPINF1*	NM_002615.6	*SC5D*	NM_006918.4
*SETBP1*	NM_015559.2	*TRIM37*	NM_015294.4	*ZMPSTE24*	NM_005857.4		

**Table 2:** A complete list of clinical features of the whole family.

	Proband	Sister	Mother	Father
Age (years)	15	14	42	41
Height (cm)	164	163	155	177
Weight (kg)	65	89	70	107
Vertebral compression fractures	1	No	No	No
Long-bone fractures	10	No	No	No
Sclerae	White	White	White	White
Subluxation of the joints	3	No	No	No
Dentinogenesis imperfecta	No	No	No	No
Hearing loss	No	No	No	No
F1 BDM (g/cm^2^)	0.604	/	/	/
F1 BDM T-score	−2.8	/	/	/
F2 BDM (g/cm^2^)	0.689	0.971	0.903	1.163
F2 BDM T-score	−2.3	0.2	−0.3	0.9
S1 BDM (g/cm^2^)	0.587	/	/	/
S1 BDM T-score	−4.6	/	/	/
S2 BDM (g/cm^2^)	0.622	0.990	0.897	0.916
S2 BDM T-score	−4.3	−0.5	−1.4	−1.6
Ca (mmol/L)	2.67 *	2.61	2.43	2.58 *
Inorganic phosphates (mmol/L)	1.39	0.84 *	1.22	1.07
Osteocalcin (µg/L)	34.0 *	18.3	7.91	3.92
vitamin D (25-OH) (nmol/L)	83	36 *	39 *	75
Creatinine (mmol/L)	12.3	4.9	13.4	15.6
Deoxypyridinoline (nM/mM of creatinine)	9.1	9.9	4.4	5.0

F1—proximal femur densitometry finding on May 20, 2021; F2—proximal femur densitometry finding on 1 September 2021; S1—spine densitometry finding on May 20, 2021; S2—spine densitometry finding on 1 September 2021; *—values outside the reference interval.

## Data Availability

The data presented in this study are available on request from the corresponding author.

## References

[1] Jovanovic M., Guterman-Ram G., Marini J.C. (2021). Osteogenesis Imperfecta: Mechanisms and Signaling Pathways Connecting Classical and Rare OI Types. Endocr. Rev..

[2] Marini J.C., Dang Do A.N., Feingold K.R., Anawalt B., Boyce A., Chrousos G., de Herder W.W., Dhatariya K., Dungan K., Grossman A., Hershman J.M., Hofland J. (2020). Osteogenesis Imperfecta. Endotext.

[3] Primorac D., Rowe D.W., Mottes M., Barisić I., Anticević D., Mirandola S., Lira M.G., Kalajzić I., Kusec V., Glorieux F.H. (2001). Osteogenesis imperfecta at the beginning of bone and joint decade. Croat. Med. J..

[4] Marom R., Rabenhorst B.M., Morello R. (2020). Management of Endocrine Disease: Osteogenesis imperfecta: An update on clinical features and therapies. Eur. J. Endocrinol..

[5] Agarwal M., Goheen M., Jia S., Ling S., White E.S., Kim K.K. (2020). Type I Collagen Signaling Regulates Opposing Fibrotic Pathways through α2β1 Integrin. Am. J. Respir. Cell Mol. Biol..

[6] Stover M.L., Primorac D., Liu S.C., McKinstry M.B., Rowe D.W. (1993). Defective Splicing of mRNA from One COL1A Allele of Type I Collagen in Nondeforming (Type I) Osteogenesis Imperfecta. J. Clin. Investig..

[7] Johnson C.V., Primorac D., McKinstry M., Rowe D.W., Lawrence J.B. (2000). Tracking COL1A1 RNA in Osteogenesis Imperfecta: Splice-defective Transcripts Initiate Transport from the Gene but are Retained within the SC35 Domain. J. Cell Biol..

[8] Fratzl-Zelman N., Wesseling-Perry K., Mäkitie R.E., Blouin S., Hartmann M.A., Zwerina J., Välimäki V.-V., Laine C.M., Välimäki M.J., Pereira R.C. (2021). Bone material properties and response to teriparatide in osteoporosis due to WNT1 and PLS3 mutations. Bone.

[9] Kim J.-M., Lin C., Stavre Z., Greenblatt M.B., Shim J.-H. (2020). Osteoblast-Osteoclast Communication and Bone Homeostasis. Cells.

[10] Guasto A., Cormier-Daire V. (2021). Signaling Pathways in Bone Development and Their Related Skeletal Dysplasia. Int. J. Mol. Sci..

[11] El-Gazzar A., Högler W. (2021). Mechanisms of Bone Fragility: From Osteogenesis Imperfecta to Secondary Osteoporosis. Int. J. Mol. Sci..

[12] Etich J., Rehberg M., Eckes B., Sengle G., Semler O., Zaucke F. (2020). Signaling pathways affected by mutations causing osteogenesis imperfecta. Cell. Signal..

[13] Rossi V., Lee B., Marom R. (2019). Osteogenesis imperfecta: Advancements in genetics and treatment. Curr. Opin. Pediatr..

[14] Hu J., Li L., Zheng W., Zhao D., Wang O., Jiang Y., Xing X., Li M., Xia W. (2020). A novel mutation in PLS3 causes extremely rare X-linked osteogenesis imperfecta. Mol. Genet. Genom. Med..

[15] Wolff L., Strathmann E.A., Müller I., Mählich D., Veltman C., Niehoff A., Wirth B. (2021). Plastin 3 in health and disease: A matter of balance. Cell. Mol. Life Sci..

[16] Schwebach C.L., Kudryashova E., Kudryashov D.S. (2021). Plastin 3 in X-Linked Osteoporosis: Imbalance of Ca2+-Dependent Regulation Is Equivalent to Protein Loss. Front. Cell Dev. Biol..

[17] Chen T., Wu H., Zhang C., Feng J., Chen L., Xie R., Wang F., Chen X., Zhou H., Sun H. (2018). Clinical, Genetics, and Bioinformatic Characterization of Mutations Affecting an Essential Region of PLS3 in Patients with BMND18. Int. J. Endocrinol..

[18] Truty R., Paul J., Ms M.K., Bs S.E.L., Olivares E., Nussbaum R.L., Aradhya S. (2019). Prevalence and properties of intragenic copy-number variation in Mendelian disease genes. Genet. Med..

[19] Lincoln S.E., Hambuch T., Zook J.M., Bristow S.L., Hatchell K., Truty R., Kennemer M., Shirts B.H., Fellowes A., Chowdhury S. (2021). One in seven pathogenic variants can be challenging to detect by NGS: An analysis of 450,000 patients with implications for clinical sensitivity and genetic test implementation. Genet. Med..

[20] Basta M., Pandya A.M. (2021). Genetics, X-Linked Inheritance. StatPearls.

[21] Mäkitie R.E., Niinimäki T., Suo-Palosaari M., Kämpe A., Costantini A., Toiviainen-Salo S., Niinimäki J., Mäkitie O. (2020). PLS3 Mutations Cause Severe Age and Sex-Related Spinal Pathology. Front. Endocrinol..

[22] Balasubramanian M., Fratzl-Zelman N., O’Sullivan R., Bull M., Peel N.F., Pollitt R.C., Jones R., Milne E., Smith K., Roschger P. (2018). Novel PLS3 variants in X-linked osteoporosis: Exploring bone material properties. Am. J. Med Genet. Part A.

[23] Wang L., Bian X., Cheng G., Zhao P., Xiang X., Tian W., Li T., Zhai Q. (2020). A novel nonsense variant in PLS3 causes X-linked osteoporosis in a Chinese family. Ann. Hum. Genet..

[24] Velthaus A., Cornils K., Hennigs J.K., Grüb S., Stamm H., Wicklein D., Bokemeyer C., Heuser M., Windhorst S., Fiedler W. (2019). The Actin Binding Protein Plastin-3 Is Involved in the Pathogenesis of Acute Myeloid Leukemia. Cancers.

[25] Schwebach C.L., Kudryashova E., Zheng W., Orchard M., Smith H., Runyan L.A., Egelman E.H., Kudryashov D.S. (2020). Osteogenesis imperfecta mutations in plastin 3 lead to impaired calcium regulation of actin bundling. Bone Res..

[26] Yorgan T.A., Sari H., Rolvien T., Windhorst S., Failla A.V., Kornak U., Oheim R., Amling M., Schinke T. (2020). Mice lacking plastin-3 display a specific defect of cortical bone acquisition. Bone.

[27] Wang W., Sarazin B.A., Kornilowicz G., Lynch M. (2018). Mechanically-Loaded Breast Cancer Cells Modify Osteocyte Mechanosensitivity by Secreting Factors That Increase Osteocyte Dendrite Formation and Downstream Resorption. Front. Endocrinol..

[28] Van Dijk F.S., Zillikens M.C., Micha D., Riessland M., Marcelis C.L., De Die-Smulders C.E., Milbradt J., Franken A.A., Harsevoort A.J., Lichtenbelt K.D. (2013). PLS3 Mutations in X-Linked Osteoporosis with Fractures. N. Engl. J. Med..

[29] Fahiminiya S., Majewski J., Al-Jallad H., Moffatt P., Mort J., Glorieux F.H., Roschger P., Klaushofer K., Rauch F. (2014). Osteoporosis Caused by Mutations inPLS3: Clinical and Bone Tissue Characteristics. J. Bone Miner. Res..

[30] Neugebauer J., Heilig J., Hosseini-Barkooie S., Ross B.C., Mendoza-Ferreira N., Nolte F., Peters M., Hölker I., Hupperich K., Tschanz T. (2018). Plastin 3 influences bone homeostasis through regulation of osteoclast activity. Hum. Mol. Genet..

[31] Roschger P., Paschalis E., Fratzl P., Klaushofer K. (2008). Bone mineralization density distribution in health and disease. Bone.

[32] Blouin S., Fratzl-Zelman N., Glorieux F.H., Roschger P., Klaushofer K., Marini J.C., Rauch F. (2017). Hypermineralization and High Osteocyte Lacunar Density in Osteogenesis Imperfecta Type V Bone Indicate Exuberant Primary Bone Formation. J. Bone Miner. Res..

[33] Kämpe A.J., Costantini A., Levy-Shraga Y., Zeitlin L., Roschger P., Taylan F., Lindstrand A., Paschalis E.P., Gamsjaeger S., Raas-Rothschild A. (2017). PLS3 Deletions Lead to Severe Spinal Osteoporosis and Disturbed Bone Matrix Mineralization. J. Bone Miner. Res..

[34] Kamioka H., Sugawara Y., Honjo T., Yamashiro T., Takano-Yamamoto T. (2004). Terminal Differentiation of Osteoblasts to Osteocytes Is Accompanied by Dramatic Changes in the Distribution of Actin-Binding Proteins. J. Bone Miner. Res..

[35] Yamauchi M., Sricholpech M., Terajima M., Tomer K.B., Perdivara I. (2019). Glycosylation of Type I Collagen. Springer Protoc. Handb..

[36] Hanagata N. (2016). IFITM5 mutations and osteogenesis imperfecta. J. Bone Miner. Metab..

[37] Al-Jallad H., Palomo T., Roughley P., Glorieux F.H., McKee M.D., Moffatt P., Rauch F. (2015). The effect of SERPINF1 in-frame mutations in osteogenesis imperfecta type VI. Bone.

[38] Komori T. (2018). Runx2, an inducer of osteoblast and chondrocyte differentiation. Histochem. Cell Biol..

[39] Liu S.-C., Sun Q.-Z., Qiao X.-F., Li X.-G., Yang J.-H., Wang T.-Q., Xiao Y.-J., Qiao J.-M. (2019). LncRNA TUG1 influences osteoblast proliferation and differentiation through the Wnt/β-catenin signaling pathway. Eur. Rev. Med. Pharmacol. Sci..

[40] Chang M.-K., Kramer I., Keller H., Gooi J.H., Collett C., Jenkins D., Ettenberg S.A., Cong F., Halleux C., Kneissel M. (2014). Reversing LRP5-Dependent Osteoporosis and SOST Deficiency-Induced Sclerosing Bone Disorders by Altering WNT Signaling Activity. J. Bone Miner. Res..

[41] Yasuda H. (2021). Discovery of the RANKL/RANK/OPG system. J. Bone Miner. Metab..

[42] Lee S.-Y., Long F. (2018). Notch signaling suppresses glucose metabolism in mesenchymal progenitors to restrict osteoblast differentiation. J. Clin. Investig..

[43] Götherström C., Walther-Jallow L. (2020). Stem Cell Therapy as a Treatment for Osteogenesis Imperfecta. Curr. Osteoporos. Rep..

[44] Dwan K., Phillipi C.A., Steiner R., Basel D. (2016). Bisphosphonate therapy for osteogenesis imperfecta. Cochrane Database Syst. Rev..

[45] Biggin A., Munns C.F. (2017). Long-Term Bisphosphonate Therapy in Osteogenesis Imperfecta. Curr. Osteoporos. Rep..

[46] Boyce A.M. (2017). Denosumab: An Emerging Therapy in Pediatric Bone Disorders. Curr. Osteoporos. Rep..

[47] Delgado-Calle J., Sato A.Y., Bellido T. (2017). Role and mechanism of action of sclerostin in bone. Bone.

[48] Paik J., Scott L.J. (2020). Romosozumab: A Review in Postmenopausal Osteoporosis. Drugs Aging.

[49] Iolascon G., Moretti A., Toro G., Gimigliano F., Liguori S., Paoletta M. (2020). Pharmacological Therapy of Osteoporosis: What’s New?. Clin. Interv. Aging.

[50] Chen G., Deng C., Li Y.-P. (2012). TGF-β and BMP Signaling in Osteoblast Differentiation and Bone Formation. Int. J. Biol. Sci..

[51] Jeleč Ž., Primorac D., Antičević D. (2019). Personalized surgery approach in severe form of osteogenesis imperfecta type III: Point of view. J. Pediatr. Orthop. B.

[52] Wang X., Thomsen P. (2021). Mesenchymal stem cell–derived small extracellular vesicles and bone regeneration. Basic Clin. Pharmacol. Toxicol..

